# Distribution of helminth eggs in environmental and stool samples of farming households along Akaki River in Addis Ababa, Ethiopia

**DOI:** 10.1186/s41182-023-00558-0

**Published:** 2023-11-24

**Authors:** Bethlhem kinfu Gurmassa, Sirak Robele Gari, Ephrem Tefera Solomon, Michaela L. Goodson, Claire L. Walsh, Bitew K. Dessie, Bezatu Mengistie Alemu

**Affiliations:** 1 Water and Health, Ethiopian Institute of Water Resources, Addis Ababa University, Addis Ababa, Ethiopia; 2 College of Health and Medical Sciences, School of Medical Laboratory Sciences, Haramaya University, Harar, Ethiopia; 3 Newcastle University Medicine Malaysia, Iskandar Puteri, Johor, Malaysia; 4 School of Engineering, Newcastle University, Newcastle Upon Tyne, Tyne and Wear UK; 5 Water and Land Resource Center, Addis Ababa University, Addis Ababa, Ethiopia

**Keywords:** Helminth, Irrigation, Soil, Wastewater, Farmers, Male

## Abstract

**Background:**

Helminth infections are a public health issue in countries with poor sanitation facilities. However, there little information on the epidemiological association between helminths in wastewater and soil samples and rates of helminth infection among farming households along the Akaki River in Addis Ababa, Ethiopia.

**Methods:**

A cross-sectional study was conducted between November 2021 and February 2022. A stratified random sampling technique was used to select farming households. The sample size for each district was determined by a proportionate allocation to the number of households. From wastewater-irrigated farms, 70 wastewater samples, 28 soil samples, and 86 farmers' stool samples were collected and analyzed for helminths. A questionnaire was used to gather ethnographic data, about farming households, whereas wastewater and soil sample analysis was used to generate quantitative data on helminth loads. The data were systematically analysed by developing themes, and bias evaluated using triangulation validation methodologies. Potential pathways to helminth infection were evaluated by measuring. Total number of helminth eggs in wastewater, soil samples and farmer's stools was investigated using Poisson regression.

**Results:**

In this study, 82.9% of wastewater samples, 57.1% of soil samples, and 18.6% of farmers' stool samples contained helminth eggs. The most prevalent helminth was *Ascaris lumbricoides* in all samples (wastewater 67%, soil 25%, and stool 10.5%), followed by hookworm (wastewater 10%, soil 21.4%, and stool 6.9%) and *Trichuris trichiura* eggs (wastewater 5.7%, soil 10.7%, and stool 1.2%). There was a positive association between the total number of helminth eggs in wastewater and soil samples with counts in farmers’ stool. The Poisson regression coefficients for wastewater and soil were, 1.63 (95% CI = 1.34–1.92) and 1.70 (95% CI = 1.39–2.01), (*p* < 0.05).

**Conclusions:**

This research has shown a clear association between the total helminth eggs in wastewater and soil samples and farmer stools along the Akaki River. Therefore, an integrated approach is essential to address the issue in this area and prevent the spread of further helminth infections.

## Background

Intestinal parasitic infections are common in tropical and subtropical regions around the globe, especially in low- and middle-income nations. Intestinal parasites, including soil-transmitted helminths, thrive due to inefficient waste management systems and inadequate environmental sanitation [1]. *Ascaris lumbricoides*, *Trichuris trichiura,* and hookworms are the most prevalent soil-transmitted helminths found worldwide. Sub-Saharan Africa, East Asia, China, India, and South America have the highest prevalence of parasitic diseases [2, 3]. Globally, over 1.5 billion people are estimated to be infected with soil transmitted helminths, which have led to an estimated 3.3 million disability-adjusted life years [5].

Helminth infections can cause major consequences in vulnerable populations, such as children, the elderly, and the immunocompromised. These consequences include dietary deficits, physical limitations, anemia, low birth weight babies, and increased perinatal mortality rates for both infants and mothers [8]. Improved hygiene and sanitation practices, such as hand washing and correct disposal of human waste, are critical in reducing helminth transmission. Antiparasitic medications can be used to control and prevent the spread of helminth infections. The World Health Organization recommends two drugs: albendazole (400 mg) and mebendazole (500 mg), both of which are efficacious, affordable, and, readily available for healthcare personnel to give to those at risk [4].

According to Minichil *et al*., the total prevalence of helminth infections in Ethiopia is estimated to be 33.35%, with the greatest prevalence in areas with poor sanitation and hygiene, such as in the farming communities alongside the Akaki River in Addis Ababa [20]. The Little and Great Akaki Rivers contribute the greatest flow (76 km) into the city. These rivers and tributaries receive huge amounts of untreated domestic and industrial wastewater flow from the city, giving the river a darkened polluted appearance.

The river water in Addis Ababa is similar to a channel transporting diluted waste from an open sewage system [20]. Despite knowing about this pollution, farmers in Addis Ababa's urban and peri-urban districts rely significantly on the polluted Akaki River to obtain water for irrigation of vegetables and for daily agricultural activities. Farmers along the river pump or manually divert untreated wastewater from the Akaki directly for vegetable irrigation. Use of contaminated river water for irrigation in vegetable planting increases the risk of soil transmitted helminthiasis. Consumption of these vegetables, leaves, farmers and their households vulnerable to direct infection with helminths [20]. The aim of the current study was, therefore, to examine the relationship between the distribution of helminth eggs in soil and wastewater along the Akaki River with human infection in farmers who irrigate with polluted wastewater.

Several studies have examined helminth infections in Ethiopia, including the capital city Addis Ababa [9, 10, 13, 14, 17, 21, 31]. The WHO (2001) has prioritized the management of helminths in preschool children, school-age children, women of reproductive age, breastfeeding women and adults in high-risk occupations, including tea pickers and miners. Few studies have looked into helminth transmission in adult males, such as agricultural workers who are at high risk of infection from eggs via soil or water or those consuming infected vegetables. Medical treatment of helminth infections does not prevent recurrent transmission of infection from chronic exposure [3]. To recurrent helminth, a preventive strategy is required. Occupational exposure is a potential cause of reinfection, where wastewater irrigation is used by farmers. In this study, an attempt was made to evaluate the distribution of soil transmitted helminth in the farming environment to identify an effective prevention regime to prevent future transmission and infection.

Even though a previous study conducted in the Akaki River [17] has documented helminth concentrations in wastewater and vegetable produced samples, there is a paucity of data that examining the epidemiological link between wastewater, soil samples and helminth infections among farmers at the same location.

The aim of this study was to determine the distribution of soil-transmitted helminths in the farming environment and assess the epidemiological link between helminth eggs in wastewater, soil samples and female farmers’ stool samples.

## Methods

### Study area

**Fig. 1:**
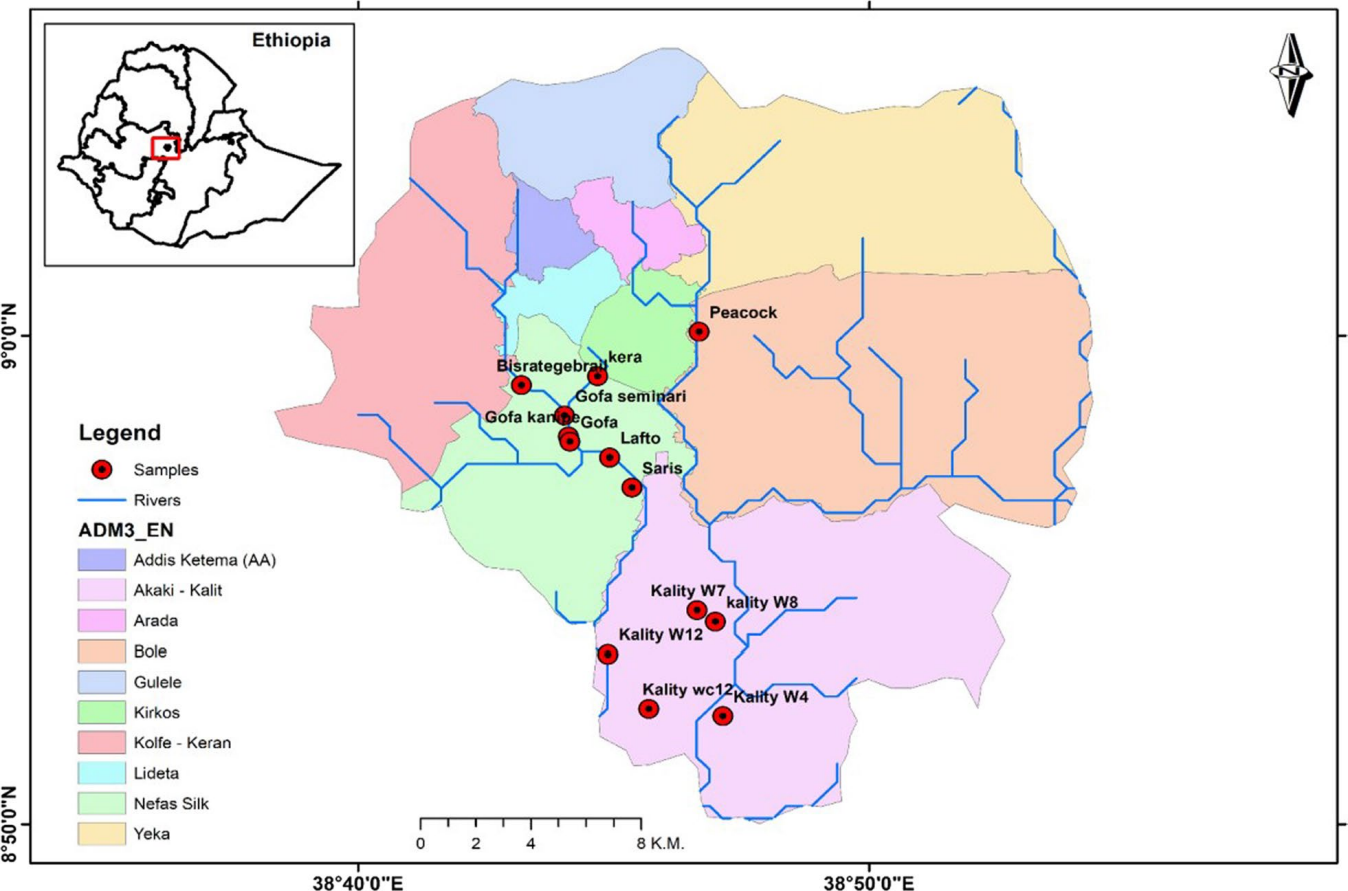
Map of the study area and the sampling sites along Akakir River Addis Ababa, Ethiopia

This study carried out in the riverside agricultural settlements of the Little and Great Akaki in Addis Ababa, Ethiopia. The population of the metropolitan area of Addis Ababa was 5,461,000 in 2023, a 4.4% increase from 2022 [11]. The city has both modern and slum neighborhoods, but the vast majority of irrigated farmlands are located close to slums [20]. Vegetable growing on agricultural land been practiced for 55–60 years [8] at various sites, known locally as Bisrategebrail, Gofa, Lafto, Saries, and Kera. They are, irrigated by the Little Akaki River. Peacock-Urael, and Akaki Kality are irrigated by the Great Akaki River (Fig. 1). The study sites are located in three sub-cities: Nifas Silk Lafto, Bole, and Akaki Kality. According to demographic forecasts from 2014 to 2017, the population of Nifas silk lafto in 2023 was 445,683, Bole was reported as 435,421, and Akaki Kality as 255,348 [12].

Vegetable crops, primarily green vegetables, such as lettuce, Ethiopian Kale and Swiss chard, are grown in these farming areas using a furrow irrigation method, which involves manually opening and closing furrows made with sandbags and coarse stones. Along the Little Akaki, in addition to furrow irrigation, flood irrigation is practiced, in which fields receive irrigation in a controlled manner by manually opening and closing a bund to flood adjacent land. The vast majority of farmers at Great Akaki farming use diesel motor pumps to collect water directly from the river and carry it to the farm via plastic pipes [20]. The soil type predominantly found at the study sites are clay, sand–clay–loam and sandy–loam.

### Study design and period

This study was a community-based cross-sectional study carried out in Addis Ababa, Ethiopia, from November 2021 to February 2022. The study evaluated, the relationship between the distribution of helminth eggs in wastewater, soil samples and stool samples of cultivators in villages that use Akaki River irrigation for vegetable cultivation.

### Population

#### Source population

The study population consisted of farming households located along the River. Since the study required stool samples, farmers’ residences had to be 3–5 m from farmland as there were no toilet facilities on or near the farmland, which would have prohibited participants providing stool samples. As a result, stool collection was conducted in farm workers’ homes, allows them to provide samples in a hygienic manner.

#### Study population

The study population consisted of male family members who worked in farming along the Akaki Riverside in Addis Ababa, Ethiopia.

### Inclusion and exclusion criteria

#### Inclusion criteria

Wastewater farmers recruited into the study met the following inclusion criteria: (a) farmers who held a sub-city identification card, and had lived in the area for more than 10 years. A 10-year timeframe was set based on the country’s land patent giver’s policy, which is a prerequisite for farmers acquire a land patent. Farmers who have secured patents are legally permitted to rent out or cultivate land themselves. This ensures that the study population comprised farmers with a legal right to cultivate the land and had a long-term affiliation to the area. (b) Only Male farmers were recruited into the study as a pilot survey showed that, male family members were in charge of a variety of chores, including land clearance and preparation, raising and forking seed beds, transplanting seed, cleaning irrigation channels, repairing dams, and watering crops. These tasks will have exposed workers to wastewater and contaminated farm soil. Since adult male farmers were most often involved in these duties, the study was limited to this group. Although it is important to note that in these areas, farming is often a family affair, with different family members having a specific role to play. (c) All male family members who participated in the farming activity needed to be available during stool collection.

#### Exclusion criteria

Exclusion criteria were: (a) daily laborers as: daily laborers working on the farm did not reside near the farmland, which made collecting stool samples challenging. Furthermore, because they do not have a permanent farm to cultivate, they go wherever they may find labor with a better income. As a result, the data acquired may be biased, because the research population may not be representative of the local farming community. (b) Farmers who use other water sources than Akaki River for irrigation were not included in the study. (c) Farmers who used more than one river water sources for irrigation were also excluded as this would make source tracking of the helminths difficult.

### Sample size

The sample size in this study was determined using the single population formula *n* = Z^2^P(1-P)/D^2^. By considering a prevalence of 7.25% in a study conducted by Legesse, 2010 [14] a marginal error of 5%, a 95% confidence interval, 5% non-respondent and Z_1-α/2_ is 1.96. Hence, the calculated sample size was 103.

### Sampling technique

**Fig. 2:**
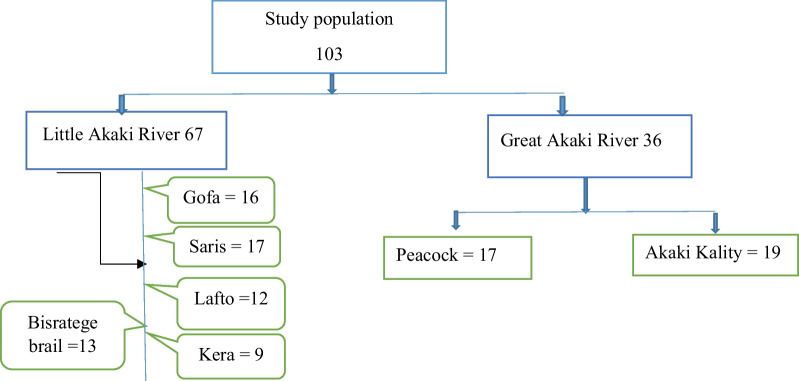
Proportional allocation of study population along Akakir River Addis Ababa, Ethiopia

A stratified random sampling method was used to sample farming households. Based on the inclusion criteria, 56 households were invited to participate in the study. The sample size of the study was (103) determined by proportional allocation to the seven study sites (Fig. 2). Following ethical approval and informed consent, out of a potential study population of 103 individuals, 86 were willing to participate and were enrolled in the study.

### Data collection

A baseline study was carried out using a structured questionnaire, interviews, and anecdotal observations. Pilot to commencing the substantive study, a pilot study was undertaken on 5% of the calculated sample size in a nearby farming area that was not included in the study to test and validate proposed methodologies.

Amendments were subsequently made to the data collection tool. To assess the distribution of helminths, a health diary, a self-reporting sheet, and laboratory stool samples were completed by participants. Data collectors were trained by the principal investigator in data collection to ensure standardization of data collection at different sites. Completed questionnaires were gathered, organized, and handed over to the researcher at the end of each working day.

### Sample collection

Samples of irrigation wastewater and soil samples at each of the seven distinct fields were collected for analysis. Each farm site's wastewater was collected in 1 L sterilized glass bottles between 8:00 and 10:00 a.m., with triplicate samples taken to ensure representative sampling had occurred. Sample points were plotted based on the farm land cover along the river, where irrigation was diverted. A grab sample was gathered from each location and analyzed. 1 L was extracted from Bisrategebrail and Kera at 11 sample points; 11 L were taken from Gofa, Lafto, and Akaki at 10 points; 10 L were obtained from Saris and 9 L from Peacock at 9 points. A total of 70 L of wastewater were collected for analysis.

During soil sample collection, superficial layers of leaves and debris was removed from the soil surface before gathering the soil samples. Samples were taken at a depth of 3 cm and stored in airtight labelled plastic bags. Each sample was divided into three sections: one from the farm entrance, two sites at the center of the farm, and then one sample was taken from the exit point of the farm. Soil samples were collected near vegetables using a random sampling techniques. Grab samples were taken at the entrance to the farm area, walking into the farm, horizontally across the field, vertically, down the field, and then down towards the farm exit. The procedure was repeated for the farm’s center and exit point. This process was carried out for each of the 4 sampling sites, yielding a total of 28 soil samples (seven samples per site).

The wastewater and farm soil samples were transported to the Ethiopian Public Health Institutes (EPHI) laboratory in an ice box and kept at 4 °C until helminth eggs were inspected. Comparisons were made between helminth loads of soil and waste water samples with WHO recommended guidelines [15].

### Sample processing for parasite detections

Soil samples were extracted into a liquid phase to detect parasites and investigated using the modified Bailenger technique. First, 100 g of soil was add to 300 ml of physiological saline solution and mixed in a vortex mixer in a 1000 ml beaker. The solution was allowed to settle overnight to facilitate the removal of vegetable soil-derived fragments and debris. The remaining sediments was then transferred into50ml centrifuge tubes and centrifuged at 1500 rpm for 3 min. A 15 ml acid/alcohol buffer solution with 5 ml ethyl acetate were added to the sediment and shaken, intermittently to expel gas [15]. The solution was then centrifuged at 2200 rpm for 3 min. The diphasic supernatant was removed, leaving about 1 ml of sediment or suspension for microscopic examination. Helminth eggs were identified using a light microscope with a magnification of 10 × and 40 × objective lenses. The number and size of the eggs were recorded. Any other intestinal parasites were also identified [15].

### Stool sample collection and examination

A clean, leak-proof, screw-cap stool cup was labelled with a unique identifier and used to gather a 2gm stool sample from each subject. The specimens were brought to the laboratory of EPHI. Following methods required by WHO, a Kato-Katz smear was prepared for each sample and examined microscopically to identify helminth eggs and quantify severity of infection based on fecal egg count [15]. The Kato-Katz slides were produced as soon as the stool specimen arrived in the laboratory.

### Data quality management

Data were entered into the statistical software, EpiData version 3.1. Data content was systematically analysed by developing themes, and validated through triangulation. The accuracy of laboratory results were controlled by undertaking a standardized process to ensure correctly labeling of samples, calibration of equipment, standard operating procedures. A data collection sheet was used to collect laboratory data, that was entered into Microsoft Excel^®^ after analysis.

### Data analysis

Data were checked for completeness and consistency then entered into EpiData Version 3.1 and exported to STATA Version 14.0 for analysis. The mean concentration and spread of eggs in environmental samples were described using descriptive data analysis. Poisson regression analysis was used to establish the association between the total number of helminth eggs in environmental samples and helminth eggs per gram of stool from farmers.

## Results

### Socio-demographic characteristics of the study participants

The majority of the respondents were middle aged 41–50 (38.4%) and over half, 54.6% (47), were illiterate. Most (65.1%) of the study participant lived with 4–6 family members and lived in 3–4 number of room house. In terms of income, 36% (31) earned between 3001 and 5000 ETB and most of them were married (89.5%) (Table 1).

**Table 1:** Socio-demographic characteristics the male farming community along Akaki River Addis Ababa, from November 2021 to February 2022

Characteristics		Frequency (%)
Sex	Male	86 (83.5)
Age	< 30	5 (5.8)
	31–40	23 (26.7)
	41–50	33 (38.4)
	> 50	25 (29.1)
Education	Educated	39 (45.3)
	Illiterate	47 (54.6)
Family size	1–3	14 (16.3)
	4–6	56 (65.1)
	> 6	16 (18.6)
Number of room	1–2	40 (16.5)
	3–4	36 (41.9)
	> 4	10 (11.6)
Income (ETB)	< 1000	15 (17.4)
	1001–3000	14 (16.3)
	3001–5000	31 (36)
	> 5000	26 (30.2)
Marta states	Single	3 (3.5)
	Married	77 (89.5)
	Divorced	4 (4.6)
	Widowed	2 (2.3)

### Distribution of parasites in environmental sample

A total of 70 wastewater, 28 agricultural soil samples, and 86 male farmers' stool samples were collected and tested for presence of helminth. Helminth was found in all the samples. The concentration of *Ascaris lumbricoides* was found to be the highest in all analyzed samples, followed by hookworm eggs and then by *Trichuris trichiura* eggs (Table 2).

**Table 2:** Distribution of helminth eggs in wastewater, soil, and stool samples from seven wastewater irrigation farming sites along Akaki River Addis Ababa, Ethiopia, from November 2021 to February 2022

Parasites	Wastewater %(*n/N*)	Soil % (*n/N*)	Farmer stool % (*n/N*)
*Ascaris lumbricoides*	67 (47/70)	25 (7/28)	10.5 (9/86)
Hookworm	10 (7/70)	21.4 (6/28)	6.9 (6/86)
*Trichuris trichiura*	5.7 (4/70)	10.7 (3/28)	1.2 (1/86)

### Distribution of helminth eggs in irrigation wastewater

The mean concentration of helminth eggs and larvae in irrigation wastewater ranged from 1.1 to 2.3 eggs per 1000 ml. One or more helminth eggs were found in 82.9% of the irrigation wastewater samples. *Ascaris lumbricoides* appear to be the most common soil-transmitted helminth in the study sites, with a mean concentration of 1.4 eggs per 1000 ml. Hookworm was the least common, with a mean concentration of 0.2 eggs per 1000 ml. The mean helminth egg concentration of irrigation wastewater in all farming sites exceeded the [15] guide value of < 1 egg per 1000 ml. The contamination was linked to untreated human or animal faeces, where farmers and neighboring villages discharge toilet waste into the river (Table 3).

**Table 3:** Mean helminth eggs distribution levels in irrigation wastewater from seven farming sites along Akaki River Addis Ababa from November 2021 to February 2022

Sample type	Farming sites						
Irrigation wastewater (*N* = 70)	Bisrategebrail (*n* = 11)	Gofa (*n* = 10)	Lafto (*n* = 10)	Saris (*n* = 9)	Kera (*n* = 11)	Peacock (*n* = 9)	Akaki (*n* = 10)
*Ascaris lumbricoides*	21	9	10	11	17	13	20
Hookworm	3	2	1	1	2	1	1
*Trichuris trichiura*	1	0	1	0	1	0	1
Total	25	11	12	12	20	14	22
Mean	2.3	1.1	1.2	1.3	1.8	1.6	2.2

Mean concentrations of helminth eggs and larvae 1000 ml^−1^

### Distribution of helminth eggs in farm soil samples

Out of the 28 soil samples collected, 16 (57.1%) tested positive for helminth eggs. The contamination rate of samples collected from different sites is presented in Table 4. The average number of helminth eggs and larvae found in all soil samples exceeded the [15] guide value of < 1 egg 100 g^−1^. Sitewise, the Akaki farming site had the highest average helminth egg concentration at 3.5 helminth eggs 100 g^−1^, while the Gofa farming site had the lowest at 1.3 helminth eggs 100 g^−1^ through (as shown in Table 4). The most commonly found egg was *Ascaris lumbricoides* (25.9%)*,* followed by hookworm (21.4%) and *Trichuris trichiura* (10.7%) (Table 4).

**Table 4:** Mean helminth eggs distribution levels in the farm soil from seven farming sites along Akaki River Addis Ababa from November 2021 to February 2022

Sample type	Farming sites						
Farm soil (*N* = 28)	Bisrategebrail (*n* = 4)	Gofa (*n* = 4)	Lafto (*n* = 4)	Saris (*n* = 4)	Kera (*n* = 4)	Peacock (*n* = 4)	Akaki (*n* = 4)
*Ascaris lumbricoides*	9	5	3	4	6	6	8
Hookworm	3	0	2	3	2	2	4
*Trichuris trichiura*	0	0	1	0	1	0	2
Total	12	5	6	7	9	8	14
Mean	3.0	1.3	1.5	1.8	2.3	2.0	3.5

The mean concentration of helminth eggs and larvae 100 g^−1^

### Load of helminth eggs in male farmers’ stool

Eighty-six participants answered the question, “Do you have helminth infection symptoms?” and provided stool samples. Out of 86 stool samples, 16 (18.6%) were positive for one of the parasites. Along the irrigation sites, (10.5%) *Ascaris lumbricoides*, (6.9%) Hookworm, and (1.2%) *Trichuris trichiura* helminths were detected. The prevalence of *Ascaris lumbricoides* infections varied significantly between sites.

**Fig. 3:**
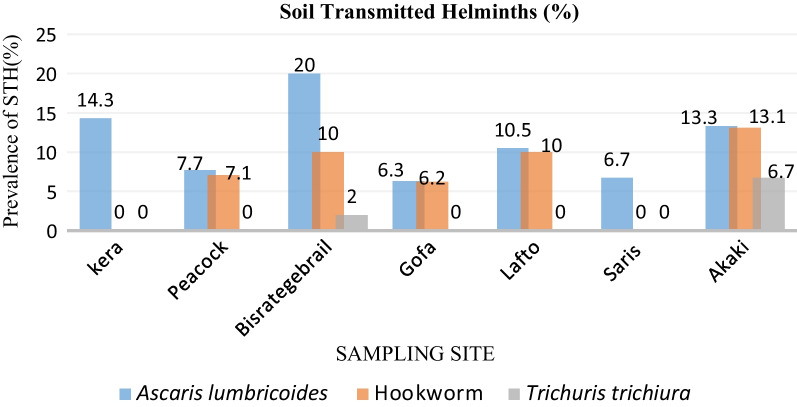
Distribution of helminths in farmers’ stools along Akakir River Addis Ababa, Ethiopia from November 2021 to February 2022

Hookworm infection was highest in the Akaki site, where 13.3% of people were infected, while *Ascaris lumbricoides* infection is highest in the Bisratgebralil site, where (20%) of individuals were infected (Fig. 3).

### Relationship between helminths egg in the irrigation wastewater, soil, and in male farmers’ stool

The total soil transmitted helminth eggs in irrigation wastewater and farm soil associated with the egg count in male wastewater vegetable farmers’ stool. The study resulted in regression coefficients of 1.63 (95% *CI* = 1.34–1.92) in the irrigated wastewater and 1.70 (95% *CI* = 1.39–2.01) in wastewater-contaminated farm soil. The association was statistically significant (*p* < 0.05) **(**Table 5**).**

**Table 5:** Relationship between helminth eggs along Akaki River the environment and male vegetable farmers’ stools in Addis Ababa from November 2021 to February 2022

Environmental sample	Coefficient	95% confidence interval	*P *value
Wastewater	1.63	1.34–1.92	0.004
Soil	1.70	1.39–2.01	0.003

## Discussion

Helminth contamination of irrigation wastewater poses a risk to human health. The eggs of helminths are highly resistant and can survive in water, soil, and plants for several months or years [18]. According to the WHO, guidelines for the use of wastewater in unrestricted agriculture should have less than 1 ova/L to ensure human health safety [15]. However, in this survey, the mean concentration of helminth egg was higher than the recommended guideline value, especially for *Ascaris lumbricoides*, with a concentration of 1.4 in wastewater and 1.5 in farm soil.

One of the main reasons for this high level of *Ascaris lumbricoides* contamination is the grey and black wastewater originating from households being connected to irrigation lines without treatment.

Of the 70 wastewater samples collected, processed, and examined, 82.9% (95% *CI* = 0.70–0.91) were determined to be infected with one or more helminth egg. The results are consistent with the earlier study conducted by Desta *et al*., 2017, in Addis Ababa where 80% of collected samples were contaminated (95% *CI* = 0.71–0.89) [17]. This finding is also higher than the findings of a study conducted in Ouagadougou, in Burkina Faso: 36.1% (95% *CI* = 0.27–0.45) [18] and in Bang B village, Vietnam: 59% (95% *CI* = 0.51–0.67) [19]. This higher level of contamination is attributed to the lack of domestic sewers and the presence of industries around the river banks without functional wastewater treatment and disposal systems, causing wastewater to drain directly into the river [22]. This deems rivers as actual sewage transporting lines [21]. This contaminated wastewater is then used for irrigation to grow vegetables, which reintroduces it into the food chain.

Among the three different helminths identified in the study, *Ascaris lumbricoides* 67% (95% *CI* = 0.55–0.79) were found to be the most prevalent parasite in the wastewater. This result is higher than the finding conducted among the main wastewater and storm water channel of Hanoi, Vietnam, where the value was 17% (95% *CI* = 0.11–0.23) [19]. *Ascarisb lumbricoides* may not be infectious soon after passing into the environment, but they can be embryonated and survive for several years under suitable conditions once deposited through irrigation [24]. The high prevalence of helminthic parasites in the study site reveals inadequate sanitation and hygiene practices within the riverside community. Individuals who are infected may not practice proper hand washing, leading to contaminated wastewater from disposed faeces [23]. The stool tested in this study also confirmed a high load of helminthic parasites in the study site. Certainly, the high load of helminthic parasites revealed in this study’s stool analysis confirms the link between farmers’ exposure to helminths and working in wastewater-irrigated farming.

Soil transmitted helminths utilize soil as a common medium for the development of helminths and eggs [24]. In this study, 57.1% (95% *CI* = 0.38–0.76) of the 28 soil samples tested positive for helminths, which is comparable to the 64.5% (95% *CI* = 0.57–0.72) rate reported by Ovutor *et al*., 2017 in Emohua, Nigeria [25] and 48% (95% *CI* = 0.39–0.75) contamination reported in a study conducted in Ouagadougou, Burkina Faso [18]. It is, considerably, higher than the 3.3% (95% *CI* = 0.1–0.13) contamination reported in a study conducted in Bazou, West Cameroon [26]. These variations in contamination rates could be attributed to factors, such as climate, topography, soil type, and the application of human face as fertilizer [1]. Soil transmitted helminth eggs can be washed deeper into the soil by irrigation and reintroduced into the farms by spreading plant parts with eggs attached [27]. A lysimeter experiment by Storey and Phillips (1985) indicated that continual irrigation could cause STH eggs to travel 2.1 cm deeper into the soil in 72 h, and egg persistence increase as they move deeper into the soil [28].

In this study, the *Ascaris lumbricoides* egg concentration in 100 g of farm soil was 1.5 egg/g, hookworm was 0.6 egg/g, and *Trichuris trichiura was* 0.1 egg/g. The relatively higher *Ascaris lumbricoides* 41 egg count in the soil sample is similar to the study conducted (38 egg count) in Cape Coast, Ghana [29]. The higher contamination of farm soil with *Ascaris lumbricoides* eggs can be described that the eggs of the parasite having an inner shell layer of lipoprotein nature which makes it more resilient to harsh environmental conditions than other helminths in the study site [29]. Moreover, a single female *Ascaris lumbricoides* lays a relatively large number of eggs (200,000 eggs/day) [29]. During the rainy season, large parts of the study site are exposed to flooding, which leads to the deposition of faecal-contaminated silt and sediment in the farmland. The washed sediment soil has a sandy composition, which is favorable for the recovery of helminth eggs in the study sites [1]. The finding of this study revealed that the prevalence of hookworm was 21.7% (95% *CI* = 0.12–0.39), and that of *Trichuris trichiura* was 10.7% (95% *CI* = 0.2–0.28). These results are comparable to a study conducted in South Africa, 21.6% (95% *CI* = 0.18–0.23), and that of *Trichuris trichiura* wa*s* 9.8% (95% *CI* = 0.7–0.16) [30].

The study site was characterized by direct exposure to wastewater and a lack of latrines, which led to defecation taking place at the edge of the agricultural field, increasing the risk of hookworm infections. The findings of the stool analysis conducted in this study indicated that load of hookworm was greater in lack of latrines coverage sites. This could be attributed to the low socioeconomic status and limited educational attainment in these areas, as suggested by previous research [29]. In addition, a study conducted in South Africa demonstrated that farmers who were exposed to excreta and wastewater through their agricultural activities had a higher incidence of hookworm infection.

This study investigated the frequency of helminth infection among Addis Ababa’s urban male vegetable farmers. The helminth load in the study was 18.6% (95% *CI* = 0.10–0.26), with *Ascaris lumbricoides* and hookworm being the most commonly discovered helminths. This finding is consistent with a community-based cross-sectional study conducted in Bibugn district, Northwest of Ethiopia, 20.9% (95% *CI* = 17.9–24.3) [31]. This finding is also comparable to a study conducted in Guangdong, China, which reported a frequency of 16.38% (95% *CI* = 0.15–0.21) [32] and the study in Chachoengsao, Thailand: 17.6% (95% *CI* = 0.10–0.24) [33]. However, it is lower than the finding of the study conducted in Zimbabwe wastewater farming, 30.2% (95% *CI* = 0.23–0.38) [33].

The use of wastewater for agricultural purposes has been associated with an increased risk of helminth infections among farmers who directly handle the wastewater. This is because the wastewater contains helminth eggs and larvae, which can infect farmers through skin contact or ingestion [29]. The duration and intensity of their contact with contaminated wastewater can further increase their risk of infection [30]. However, it is not only the risk of helminth infection that increases with wastewater irrigation—exposure to farm soil and vegetables also needs to be considered [30].

In this study, the statistically significant (*p* < 0.05) positive regression coefficient suggests that there was a positive association between total number of STH eggs in wastewater, farm soil, and male vegetable farmers’ stool. This coefficient indicates that an increase in total number of STH eggs in wastewater and farm soil was associated with an increase in the number of cases of STH infection among male vegetable farmers. The male farmers and their family members who utilize wastewater irrigation are 63% chances of developing STH infections.70% of these farmers were exposed to STH infection being working in wastewater-contaminated farm soil. This epidemiological prevalence highlights occupational exposure to wastewater and contaminated soil as a significant contributing factor to the prevalence of STH infections among wastewater vegetable farmers. The STH infection in wastewater, frequency of exposure, immunological status, and protective coverings could be attributed to influences for the transformation of STH parasites from the environments to the wastewater vegetable farmers [36].

From the survey, factors that were found to increase farmers’ infection prevalence included all daily fieldwork and agricultural activities, such as irrigation, transplanting, weeding, and harvesting, which were done with bare hands and feet. In addition, farmers feed, chew khat, and wash farm work materials, their hands, feet, and the harvested vegetables with the irrigated wastewater. Thus, this contamination could serve as a source for the re-introduction of eggs into the irrigation water channels.

The study demonstrated the epidemiological link between wastewater irrigation and helminth infection among farmers. The detailed collection of environmental samples along the river line provides valuable information for environmental and community health surveillance. However, the study's limitations, such as its focus on only leafy vegetable farmers and the absence of helminth infection identification in the vegetable market sellers, may impact the generalizability of the findings.

## Conclusions

The overall mean concentration of helminth egg loads was found to be above the WHO's unrestricted irrigation standard in the study sites. *Ascaris lumbricoides* was the most common helminth egg found, followed by hookworm and *Trichuris trichiura*. The results suggest that irrigation wastewater in eight out of ten examined sites was contaminated with helminth eggs, which could be a potential source of infection for farmers and vegetable consumers. More than half of the soil samples tested were found to be contaminated with helminth. In addition, one out of five examined male farmers who use wastewater irrigation was found to be infected with soil-transmitted helminths. Irrigating farmland with helminth-contaminated wastewater can lead to contamination of the soil, vegetable, and humans.

In general, the variation of helminth prevalence rates may be explained by individual factors, such as the ability to harbor the intensity of helminth infection, as well as proactive and environmental factors. However, for STH-positive farmers, they need to take anthelmintic medication, but it is recommended to seek medical treatment immediately to prevent further health complications. Moreover, good hygiene practices, such as washing hands frequently and proper disposal of faecal waste, to prevent the spread of diseases is recommended.

To prevent this public health threat, an integrated set of approaches is necessary, such as adequate wastewater treatment, vegetable restriction, and appropriate wastewater-irrigation techniques. By strengthening policies and enforcing laws on sewage waste discharge, both industries and the community can improve the Akaki River. This will reduce the prevalence of STH in farming environments. Farmers should practice good hygiene, such as washing their hands with soap and water before eating or handling food, and use protective equipment, such as gloves and boots, when working with wastewater. In addition, the government deworming programme must include this exposed community.

Furthermore, comprehensive health education programmers should be implemented among the farming community and general population to raise awareness of the dangers of working and living in a helminth-contaminated environment and ensure effective sanitation practices concerning hand washing and personal hygiene, as well as vegetable preparation. Further research may be necessary to understand the risk factors associated with helminth prevalence, further health complications, and household cross-contamination.

### Operational definitions

**Farmers:** those communities cultivate vegetables along the Akaki River side.

**Wastewater:** untreated Little and Great Akaki River.

**Permanent:** farmers have lived along the Akaki River side for more than 10 years.

**Environmental samples:** the study samples were taken from wastewater and soils along the Akaki Riverside farm.

## Data Availability

The data used in this manuscript are not publicly available due to ongoing analyses. Data presented in this study are available from the corresponding author upon reasonable request.

## Abbreviations

STH: Soil Transmitted Helminths
WHO: World Health Organization
EPHI: Ethiopian Public Health Institute
